# Occurrence of Microplastics and Potentially Toxic Elements in Red Sea Crab (*Portunus pelagicus*) and Squid (*Uroteuthis* spp.): Contamination and Food-Safety Concerns

**DOI:** 10.3390/toxics14100888

**Published:** 2026-10-02

**Authors:** Omar M. L. Alharbi

**Affiliations:** Department of Biology, College of Science, Taibah University, Madinah 42353, Saudi Arabia; omharbi@taibahu.edu.sa

**Keywords:** microplastics, polyethylene, potentially toxic elements, seafood contamination, dietary exposure, human health risk, Red Sea

## Abstract

Microplastics (MPs) and potentially toxic elements (PTEs) are co-occurring marine contaminants, yet integrated assessments of Red Sea seafood remain limited. This study evaluated MP contamination, PTE bioaccumulation, and dietary health risks in *Portunus pelagicus* (*n* = 281) and *Uroteuthis* spp. (*n* = 159) from Yanbu, Rabigh, and Umluj. A standardized subset of 180 specimens was examined for MPs using stereomicroscopy, yielding 508 suspected particles. All 508 particles were examined by FTIR, of which 260 (51.2%) were spectroscopically confirmed as polyethylene (PE), while 248 (48.8%) remained unconfirmed. Crabs showed approximately 10–89-fold higher suspected-particle burdens than squid (0.742–1.683 vs. 0.008–0.167 particles/g wet weight). Eleven PTEs were quantified in edible muscle by ICP-MS. Squid contained approximately 17–24-fold higher Fe and 5–7-fold higher Cd, whereas crabs contained 11–13-fold higher total As and higher Zn. Non-carcinogenic risk remained below the conventional threshold (HI < 1) across all scenarios, whereas estimated arsenic-related carcinogenic risk exceeded 1 × 10^−4^ in some crab-consumption scenarios under the assumed 7% inorganic-As fraction. Exploratory MP–PTE associations were strongly influenced by species identity and cannot establish MP-mediated PTE transfer.

## 1. Introduction

Microplastic (MP) contamination and the accumulation of potentially toxic elements (PTEs) in marine ecosystems are important environmental and food-safety concerns. MPs (plastic particles < 5 mm) occur in various morphological forms, including fibers, fragments, films, foams, and beads, and may consist of different polymer types, commonly including polyethylene (PE), polypropylene (PP), polyethylene terephthalate (PET), and polystyrene (PS). MPs are widely reported in marine biota and seafood, creating a potential pathway for dietary exposure [1,2,3]. PTEs such as As, Cd, and Pb can also accumulate in seafood tissues and contribute to non-carcinogenic or carcinogenic risk under sustained dietary exposure [4,5,6]. Because MPs and PTEs co-occur in the same environments and organisms, integrated assessment is useful for characterizing co-exposure and for testing whether the two contaminant classes covary across species and sites. MPs can sorb PTEs from seawater onto their polymer surfaces and deliver them to organisms upon ingestion, leading to the hypothesis that MPs may act as vectors for metal transfer; however, the quantitative importance of this vector role in natural food webs remains debated, and co-occurrence alone does not establish vector-mediated transfer. It is also important to distinguish the quantitative health-risk assessment that can be performed for PTEs, using established oral reference doses and cancer slope factors, from the exposure context provided by MP data, for which no validated dose–response framework for seafood consumption currently exists.

Crustaceans and cephalopods are ecologically and economically important components of marine food webs, but their exposure pathways differ. The blue swimming crab, *Portunus pelagicus*, is a benthic-associated omnivore and scavenger that has substantial contact with sediments and benthic prey, whereas *Uroteuthis* spp. are mobile predatory cephalopods that forage primarily in the water column. These ecological differences can produce contrasting patterns of MP exposure and retention. Previous studies have documented MP contamination in *Portunus pelagicus* from the Persian Gulf and the Gulf of Thailand, with fibers or fragments frequently reported and several common packaging- and fishing-related polymers identified [4,7]. However, integrated assessments of MPs and PTEs in these taxa from the Saudi Arabian Red Sea remain limited.

The Red Sea is a unique semi-enclosed basin experiencing increasing anthropogenic pressure from coastal development, industrial activity, shipping, and desalination [3,8,9]. Along the Saudi Arabian coast, Yanbu and Rabigh are major industrial centers, whereas Umluj is comparatively less industrialized, providing contrasting coastal settings for assessing contamination. Despite the commercial importance of *Portunus pelagicus* and *Uroteuthis* spp. in regional fisheries, integrated MP–PTE assessments in these organisms remain scarce.

This study aimed to provide an integrated assessment of MP contamination, PTE bioaccumulation, and PTE-related human health risks in *Portunus pelagicus* and *Uroteuthis* spp. collected from Yanbu, Rabigh, and Umluj. Specifically, the study (i) characterized the abundance, tissue distribution, morphology, representative particle size, and polymer composition of suspected MPs in gill and digestive tissues; (ii) quantified eleven PTEs (Zn, Fe, As, Cd, Cu, Co, Ni, Mn, Cr, Pb, and Hg) in edible muscle; (iii) compared contaminant profiles between species and among locations; and (iv) evaluated dietary exposure and associated non-carcinogenic and carcinogenic risks for adult and child consumers, and (v) explored covariation between MP abundance and PTE concentrations across species–site groups as an organism-level, exploratory test of whether the two contaminant classes are coupled beyond species identity. By integrating MP and PTE measurements within the same species and geographic framework, this study establishes a comparative baseline for Red Sea seafood contamination and identifies species- and site-specific patterns relevant to seafood safety and environmental monitoring.

## 2. Materials and Methods

### 2.1. Study Area and Sampling Design

**Figure 1:**
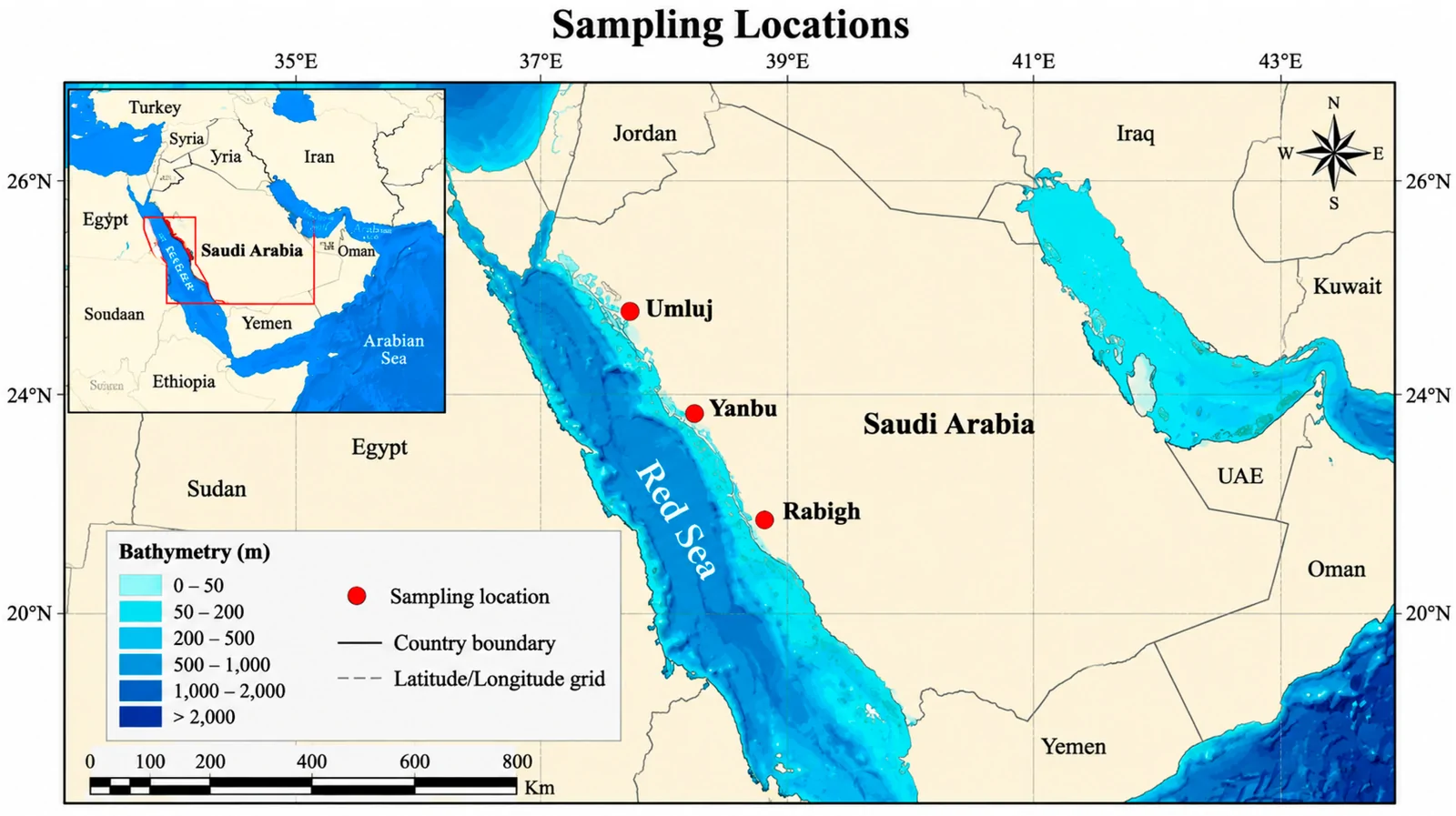
Sampling locations along the Saudi Arabian Red Sea coast: Umluj, Yanbu, and Rabigh.

The study was conducted at three locations along the Saudi Arabian Red Sea coast representing contrasting coastal settings: Yanbu (24.09° N, 38.06° E), Rabigh (22.80° N, 39.03° E), and Umluj (25.02° N, 37.27° E) (Figure 1). Yanbu and Rabigh are industrialized coastal areas characterized by petrochemical, shipping, desalination, and other industrial activities, whereas Umluj was selected as a site with comparatively lower levels of industrial development [8]. Commercially harvested blue swimming crab (*Portunus pelagicus*) and needle squid (*Uroteuthis* spp.) were obtained from local fishing vessels operating in coastal waters adjacent to the study locations between September 2024 and January 2025. A total of 440 specimens were examined, comprising 281 crabs and 159 squid. Samples included 95 crabs and 54 squid from Yanbu, 93 crabs and 53 squid from Rabigh, and 93 crabs and 52 squid from Umluj. Specimens were transported under chilled conditions and processed within 24 h. Edible muscle was dissected for PTE determination, whereas gill and digestive tissues were collected for MP analysis. Because specimens were obtained from commercial fishing vessels, the sample reflects the size range retained by the fishery, and individual ages could not be determined. Specimens were assigned to each site based on the landing port, and the fishing vessels’ operating areas were confirmed with the fishers at the time of sampling to ensure that the catch originated from the intended coastal waters. The coordinates shown in Figure 1 represent the general sampling areas rather than specimen-specific capture locations; exact specimen-level GPS coordinates and distances from shore were not recorded during the commercial fishing operations. No site-specific measurements of water, sediment, prey, industrial effluents, or other environmental matrices were conducted in the present study; therefore, the characterization of Yanbu and Rabigh as industrialized coastal settings is contextual, based on documented regional petrochemical, shipping, and desalination activities [3,6] and previous evidence of heavy-metal contamination in coastal sediments at Yanbu [8] rather than a direct measure of local contaminant exposure during the present study.

### 2.2. MP Extraction and Isolation from Crabs and Squid

MPs were extracted from gill and digestive tissues using an alkaline digestion and density-separation procedure adapted from established protocols for biological samples. A fixed 2.0 g wet-weight (ww) portion of each tissue was dissected using clean stainless-steel instruments and transferred to a pre-cleaned glass vessel. Samples were digested with 10% potassium hydroxide (KOH, analytical reagent grade, ≥85% purity; Sigma-Aldrich, St. Louis, MO, USA) at 50 °C for approximately 24 h, or until visible biological material was sufficiently digested [10,11]. Following digestion, density separation was performed using 600 mL of filtered saturated NaCl solution (NaCl, analytical grade, ≥99.5% purity; Sigma-Aldrich, St. Louis, MO, USA) (density approximately 1.2 g/mL) [12]. This approach preferentially recovers lower-density polymers, including PE and PP, whereas particles with densities greater than that of the separation medium, such as polyethylene terephthalate (PET) and polyvinyl chloride (PVC), may be incompletely recovered. Consequently, the use of NaCl may have led to an underestimation of the abundance of higher-density microplastics. Because polymer-specific recovery tests were not performed in the present study, the magnitude of this potential underestimation could not be quantified. After phase separation, the floating fraction containing suspected MPs was carefully recovered and vacuum-filtered through 0.45 µm cellulose nitrate membrane filters (47 mm diameter; Whatman/Cytiva, Little Chalfont, Buckinghamshire, UK) using a glass filtration apparatus. Digestion and separation vessels and the filtration apparatus were rinsed with filtered ultrapure water (18.2 MΩ·cm), and the rinsates were passed through the same membrane to maximize particle recovery. Filters were transferred immediately to clean, covered glass Petri dishes and stored under dry, contamination-controlled conditions until microscopic and instrumental examination. Because a fixed 2.0 g tissue mass was processed, MP abundance was normalized to tissue wet weight and expressed as particles/g ww [11,13,14,15]. The use of higher-density separation media (e.g., ZnCl_2_ or NaI solutions) is recommended for future work.

### 2.3. MP Identification and Characterization

The retained particles were screened systematically using a digital binocular stereomicroscope (10×–100× magnification; Leica Microsystems, Wetzlar, Germany), and the entire filter surface was examined to minimize particle omission and double counting. Suspected MPs were photographed, counted, and classified by morphology, color, and size. Morphology was assigned using predefined criteria: fibers were defined as elongated particles with a length-to-width ratio greater than 3:1; fragments as irregular or angular particles; and films as thin, flat, sheet-like particles. Particle size was measured as the longest dimension from calibrated images using ImageJ software (version 1.53f; U.S. National Institutes of Health, Bethesda, MD, USA). Representative suspected particles were further characterized by field-emission scanning electron microscopy coupled with energy-dispersive X-ray spectroscopy (FESEM-EDS; JSM-IT800, JEOL, Tokyo, Japan), with selected particles prepared using a JEOL Dll-29030SCTR coater (JEOL, Tokyo, Japan). Polymer identity was assessed by FTIR spectroscopy using a Nicolet™ iS™5 FTIR Spectrometer (Thermo Fisher Scientific, Waltham, MA, USA) for all 508 microscopically identified suspected particles. The obtained spectra were evaluated by comparison with reference spectra and diagnostic absorption bands for polymer identification. Only spectra of sufficient quality and interpretability were used for polymer identification. MP abundance was normalized to tissue wet weight and expressed as particles/g ww; particle distributions were summarized by tissue, morphology, and approximate size, with color recorded for representative particles; owing to the small particle numbers in several species–site groups, color and size summaries are qualitative. A formal lower particle-size detection limit was not experimentally validated for the stereomicroscope configuration used. Accordingly, the 0.45 µm membrane pore size should not be interpreted as the analytical detection limit; particles below the practical optical resolution of the stereomicroscopic procedure may have been underestimated. Successful FTIR identification was restricted to particles that yielded spectra of sufficient quality for reliable interpretation [15,16,17,18].

### 2.4. Quality Assurance and Contamination Control for Microplastics

Strict contamination-control procedures were applied throughout dissection, digestion, density separation, filtration, and particle characterization. Glassware was preferentially used; stainless-steel instruments were cleaned between specimens with filtered ultrapure water (18.2 MΩ·cm) and 70% ethanol [prepared using HPLC-grade absolute ethanol (≥99.9%; Sigma-Aldrich, St. Louis, MO, USA)]; and aqueous solutions and rinsing water were filtered before use. Samples, digestion vessels, filtration units, and recovered filters were kept covered whenever practicable to minimize airborne contamination. Procedural blanks containing all reagents but no biological tissue were processed alongside analytical samples and examined using the same microscopic procedure. Total particle abundance was derived from systematic microscopic counts and is therefore reported as microscopy-based suspected-particle abundance, with polymer identification based only on particles yielding interpretable FTIR spectra [15,17,18]. Procedural blanks (*n* = 3) yielded no suspected MP particles (mean = 0 particles/filter); therefore, no blank correction was applied to the sample MP counts.

### 2.5. Assessment of Microplastic Contamination Levels

To evaluate relative spatial loading of microplastics, a contamination factor (CF), degree of contamination (CD), and relative pollution load index (rPLI) were calculated separately for each species using an index framework adapted from Tomlinson et al. [19]. Because the original PLI framework was developed for chemical contaminants rather than microplastics, the resulting classifications are descriptive and should not be interpreted as validated ecological-risk thresholds for MPs. A CF of 1 means the site matches the reference concentration, while values above 1 indicate progressively greater contamination relative to that baseline. The contamination factor for each site was calculated as (Equation (1)):

(1) $$\mathrm{CFi}=\frac{\mathrm{Ci}}{C_{0}}$$

where Ci is the mean MP abundance at site i, and C_0_ is the reference concentration (lowest species-specific mean). No minimum particle-count threshold was applied to the background; C_0_ was defined as the lowest species–site group means. Because each group mean integrates 30 specimens, a low C_0_ does not imply that every specimen contained particles; specimens with zero detected particles were observed across several tissue–site combinations. For example, all squid gill samples at Umluj and Rabigh contained no detected particles. Because no established ecological baseline for microplastic contamination exists for *Portunus pelagicus* or *Uroteuthis* spp. in the Red Sea, the lowest species-specific mean MP abundance among the sampled sites was used as the reference concentration (C_0_). CF, CD, and rPLI were calculated separately for each species and were interpreted only as descriptive measures of relative spatial loading. The degree of contamination for each species was calculated as the sum of site-specific CF values (Equation (2)):

(2) CD= ∑CFi

where n is the number of sampling sites (*n* = 3). The rPLI for each species was calculated as the geometric mean of the site-specific CF values (Equation (3)):

*rPLI* = (*CF*_1_ × *CF*_2_ × *CF*_3_)^1/3^ (3)

CF, CD, and rPLI were used only to standardize spatial loading within each species. Because each species was referenced to its own lowest mean abundance, these indices were not used to compare absolute contamination between crab and squid or as validated ecological-risk thresholds [20,21].

### 2.6. PTE Determination by ICP-MS

Edible muscle tissues were freeze-dried and homogenized before elemental analysis. Approximately 0.50 g dry weight of homogenized tissue was digested with 12 mL concentrated HNO_3_ (65–69%; Sigma-Aldrich, St. Louis, MO, USA) and 6 mL H_2_O_2_ (30% *v*/*v*; Sigma-Aldrich, St. Louis, MO, USA) at 140 ± 5 °C for 1.5–2 h. Following digestion, samples were filtered and diluted to a final volume of 50 mL with ultrapure water (18.2 MΩ·cm) [3,8,22].

Eleven PTEs—Zn, Fe, As, Cd, Cu, Co, Ni, Mn, Cr, Pb, and Hg—were quantified using inductively coupled plasma mass spectrometry (ICP-MS; Agilent 7500 Series, Agilent Technologies, Inc., Santa Clara, CA, USA). Helium collision-cell mode was used to minimize polyatomic interferences. Instrument calibration was performed using multi-element standards (certified multi-element calibration standard solutions; Sigma-Aldrich, St. Louis, MO, USA), with calibration coefficients (R^2^ > 0.999). Analytical accuracy was evaluated using certified reference material DORM-4 (fish protein; National Research Council Canada, Ottawa, ON, Canada), with recoveries ranging from approximately 92% to 105%. Concentrations were converted using measured tissue moisture content and expressed as mg/kg wet weight (ww) to facilitate comparison with food-safety standards and dietary-exposure calculations [8,22]. For each species–site group, edible muscle from 30 individual specimens was analyzed by ICP-MS; consequently, the mean ± SD values reflect between-specimen variability (*n* = 30 specimens per species–site group).

### 2.7. Calculation of Metal Pollution Index

The Metal Pollution Index (MPI) was calculated to provide an integrated measure of the overall potentially toxic element burden in the edible muscle of crabs and squid at each sampling location. The MPI was calculated as the geometric mean of the concentrations of the elements included in the analysis (Equation (4)):

*MPI* = (*C*_1_ × *C*_2_ × *C*_3_ … *C_n_*)^1/n^ (4)

where (C_n_) is the concentration (mg/kg wet weight) of the (nth) element in the edible tissue, and (n) is the total number of elements included in the calculation. Higher MPI values indicate a greater overall elemental burden and were used for relative comparison among species and sampling locations. MPI was treated as a comparative contamination index rather than as a toxicological or human health risk threshold [23].

### 2.8. Human Health Risk Assessment Models

Human health risk assessment (HHRA) estimates the magnitude and probability of adverse health effects in humans exposed to contaminants in environmental media. Dietary exposure to PTEs was evaluated under four consumer scenarios: adult females (BW = 55 kg; ED = 70 years; FIR = 7 g/day), children aged 6–12 years (BW = 25 kg; ED = 10 years; FIR = 3.5 g/day), children aged 2–6 years (BW = 15 kg; ED = 6 years; FIR = 2 g/day), and high-consumption adults (BW = 60 kg; ED = 70 years; FIR = 35 g/day) [24,25,26]. The high-consumption adult scenario was treated as a sensitivity scenario. Exposure frequency (EF) was fixed at 365 days/year.

Estimated daily intake (EDI; mg/kg body weight/day) was calculated as (Equation (5)):

(5) $$\mathrm{EDI}=\;\frac{C\;\times \mathrm{FIR}}{\mathrm{BW}}$$

where C is the mean PTE concentration in edible muscle (mg/kg wet weight), FIR is the seafood ingestion rate (kg/day), and BW is body weight (kg) [27,28].

### 2.9. Non-Carcinogenic Risk

Target hazard quotient (THQ) is defined as the ratio of intake (chronic daily intake) to the oral reference dose, with THQ < 1 indicating a low likelihood of appreciable non-carcinogenic effects and THQ > 1 indicating a potential concern [27,29,30]. Non-carcinogenic risk was evaluated using the THQ (Equation (6)):

(6) $$\mathrm{THQ}=\;\frac{\mathrm{EDI}}{\mathrm{RfD}}$$

where RfD is the oral reference dose for the respective element. The RfDs applied were 6 × 10^−5^ mg/kg/day for inorganic arsenic (iAs), 1 × 10^−3^ mg/kg/day for Cd, and 1.5 mg/kg/day for Cr(III). The 2025 U.S. EPA IRIS assessment supports an oral RfD of 6 × 10^−5^ mg/kg/day for iAs [31]. A THQ < 1 indicates a low likelihood of appreciable non-carcinogenic effects under the specified exposure scenario, whereas THQ ≥ 1 indicates potential concern [29,32]. The Hazard Index (HI) was calculated as the sum of the individual THQs; HI < 1 was interpreted as a low likelihood of combined non-carcinogenic effects under the modeled assumptions [32,33].

(7) $$\mathrm{HI}=\sum {\mathrm{THQ}}_{i}$$

where THQi represents the individual THQ for each PTE included in the cumulative assessment. Because the EDI in Equation (5) already represents a chronic daily intake under the assumption of daily consumption throughout the exposure duration, no additional averaging time was required for the non-carcinogenic assessment.

### 2.10. Inorganic Arsenic and Carcinogenic Risk

Because arsenic speciation was not directly determined, inorganic arsenic was conservatively assumed to constitute 7% of measured total arsenic [34,35,36]. The estimated iAs concentration was calculated using Equation (8).

*C_*i*_As* = *C total As* × 0.07 (8)

The lifetime average daily dose (LADD) of iAs was calculated as (Equation (9)):

(9) $${\mathrm{LADD}}_{i}\mathrm{As}=\;\frac{C_{i}\mathrm{As}\times \mathrm{EF}\times \mathrm{ED}\times \mathrm{FIR}}{\mathrm{BW}\times \mathrm{ATc}}$$

where C_i_As is the estimated inorganic arsenic concentration (mg/kg wet weight), FIR is the ingestion rate (kg/day), EF is exposure frequency (365 days/year), ED is exposure duration (years), BW is body weight (kg), and ATc is the carcinogenic lifetime averaging time [30,36,37]. A fixed lifetime averaging time of 70 years was applied to all consumer groups:

ATc = 70 × 365 = 25,550 days

Carcinogenic risk was subsequently calculated as (Equation 10):

*CR* = *LADD_*i*_As* × *CSF* (10)

where CSF is the 2025 U.S. EPA IRIS oral cancer slope factor for inorganic arsenic, 32 (mg/kg-day)^−1^ [31]. Because iAs was estimated from total arsenic rather than measured directly by speciation analysis, the resulting CR values are assumption-dependent screening estimates rather than direct measurements of carcinogenic exposure (Table 1). The combined oral CSF of 32 (mg/kg-day)^−1^ applies to the low-dose region, which displays an approximately linear dose–response relationship (intakes < 0.2 µg/kg-day). All LADD values estimated in the present study, including the maximum of approximately 0.03 µg/kg-day, fall within this low-dose region [31].

### 2.11. Statistical Analysis

All statistical analyses were performed using IBM SPSS Statistics, version 31.0.2.0 (IBM Corp., Armonk, NY, USA). Statistical analyses summarized MP abundance and PTE concentrations across species and sampling locations. For MP data, the discrete distribution and excess zeros, particularly in squid, supported nonparametric inference [38,39,40]. Site effects within each species were evaluated using Kruskal–Wallis tests; significant site effects were followed by pairwise rank-based comparisons with Holm adjustment for multiple testing [41]. Crab-versus-squid comparisons at each site were analyzed using Mann–Whitney U tests, with rank-biserial correlation reported as the effect size [42,43]. Paired differences in particle counts between gill and digestive tissues were evaluated using two-sided Wilcoxon signed-rank tests. Pairs with zero differences were excluded from the ranking procedure (Wilcoxon zero-difference convention), and *p*-values were calculated using the asymptotic normal approximation with tie correction and without continuity correction. Statistical significance was set at *p* < 0.05. PTE concentrations were summarized descriptively as mean ± SD, with each mean and SD calculated from the 30 individual specimens analyzed per species–site group; however, the specimen-level concentration files were not retained in the analytical laboratory’s archive, and only group-level summaries could be recovered for the present analysis. Consequently, the specimen-level observations required for valid inferential testing of PTE differences were unavailable, and no inferential *p*-values are reported for PTE comparisons. To explore potential organism-level relationships between the two contaminant classes, Spearman rank correlations were computed between total MP abundance (gill plus digestive tissues; particles/g ww) and each muscle PTE concentration across the six species–site group means (*n* = 6); species-adjusted associations were then examined using partial rank correlations and multiple linear regression with MP abundance as the response variable and species (crab vs. squid) and individual PTE concentrations as predictors. Because MPs and PTEs were quantified in different tissues, these analyses reflect only organism-level covariation; moreover, given the small number of groups (*n* = 6) relative to the number of predictors, they were treated as exploratory and descriptive rather than confirmatory [4,44,45].

## 3. Results

### 3.1. Microplastic Abundance, Tissue Distribution, and Species Differences

The MP dataset comprised 30 specimens per site for each species, with 4.0 g wet weight (ww) of tissue analyzed per specimen (2.0 g gill and 2.0 g digestive tissue). Total suspected-particle abundance in *Portunus pelagicus* was 0.742 ± 0.413 particles/g ww at Umluj, 1.608 ± 0.659 particles/g ww at Rabigh, and 1.683 ± 0.557 particles/g ww at Yanbu. A total of 508 suspected particles were counted across the 180 analyzed specimens, comprising 484 particles in crabs and 24 particles in squid. FTIR examination was performed on all 508 microscopically identified suspected particles. Of these, 260 particles (51.2%) were spectroscopically identified as PE, whereas 248 particles (48.8%) could not be reliably confirmed by FTIR. Accordingly, polymer identification was limited to spectroscopically confirmed particles, and unconfirmed particles were retained as microscopy-based suspected particles. Corresponding values in *Uroteuthis* spp. were substantially lower, at 0.008 ± 0.046, 0.025 ± 0.076, and 0.167 ± 0.240 particles/g ww, respectively (Table 2). Thus, crab MP abundance was approximately 89-fold, 64-fold, and 10-fold higher than that of squid at Umluj, Rabigh, and Yanbu, respectively. The calculated CF, CD, and rPLI values are presented in Table 3. For squid, Umluj had the lowest mean MP abundance (C_0_ = 0.0083 particles/g ww) and was therefore used as the species-specific reference. Because this reference value was very low, the resulting CF and rPLI values were highly sensitive to small absolute differences in MP abundance among sites. Consequently, the relatively high index values obtained for squid should be interpreted cautiously as descriptive measures of relative spatial variation rather than as evidence of severe microplastic contamination.

Suspected-particle abundance differed significantly among crab sites (Kruskal–Wallis H = 35.51, *p* = 1.95 × 10^−8^). Pairwise comparisons with Holm adjustment showed significantly higher abundance at Rabigh than at Umluj (adjusted *p* = 2.64 × 10^−6^) and at Yanbu than at Umluj (adjusted *p* = 1.77 × 10^−7^), whereas Rabigh and Yanbu did not differ significantly (adjusted *p* = 0.710). A significant site effect was also detected in squid (H = 18.45, *p* = 9.85 × 10^−5^). Squid abundance was significantly higher at Yanbu than at Umluj (adjusted *p* = 0.000832) and Rabigh (adjusted *p* = 0.00588), whereas Umluj and Rabigh did not differ significantly (adjusted *p* = 0.313). At each site, suspected-particle abundance was significantly higher in crabs than in squid (Mann–Whitney U = 896.5, 898.5, and 897.0 for Umluj, Rabigh, and Yanbu, respectively; all *p* ≤ 2.12 × 10^−11^), with very large rank-biserial correlations (0.992–0.997).

Within crabs, digestive-tissue particle counts were significantly higher than paired gill counts at Umluj, Rabigh, and Yanbu (Wilcoxon signed-rank tests, *p* = 3.52 × 10^−6^, 3.07 × 10^−6^, and 2.81 × 10^−6^, respectively). In squid, digestive-tissue counts were significantly higher than gill counts only at Yanbu (*p* = 0.000632), whereas differences were not significant at Umluj (*p* = 0.317) or Rabigh (*p* = 0.0833).

### 3.2. Microplastic Morphology, Size, and Polymer Composition

**Figure 2:**
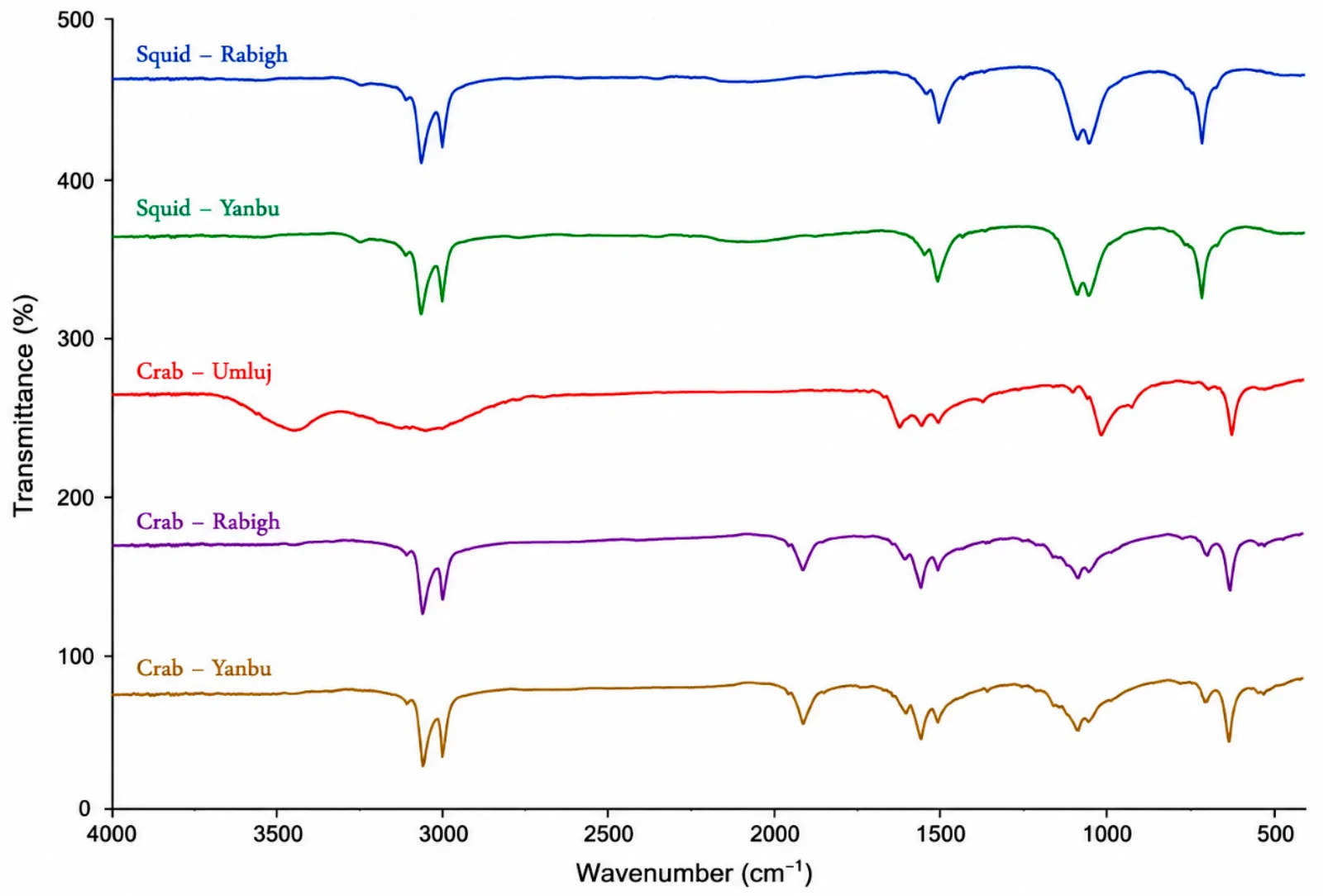
Representative FTIR spectra of ten particles spectroscopically identified as polyethylene (PE). A total of 260 of the 508 microscopically identified suspected particles (51.2%) were confirmed as PE by FTIR, while 248 particles remained spectroscopically unconfirmed. The ten spectra shown illustrate representative PE spectral profiles obtained from crab and squid samples collected from Rabigh, Yanbu, and Umluj.

**Figure 3:**
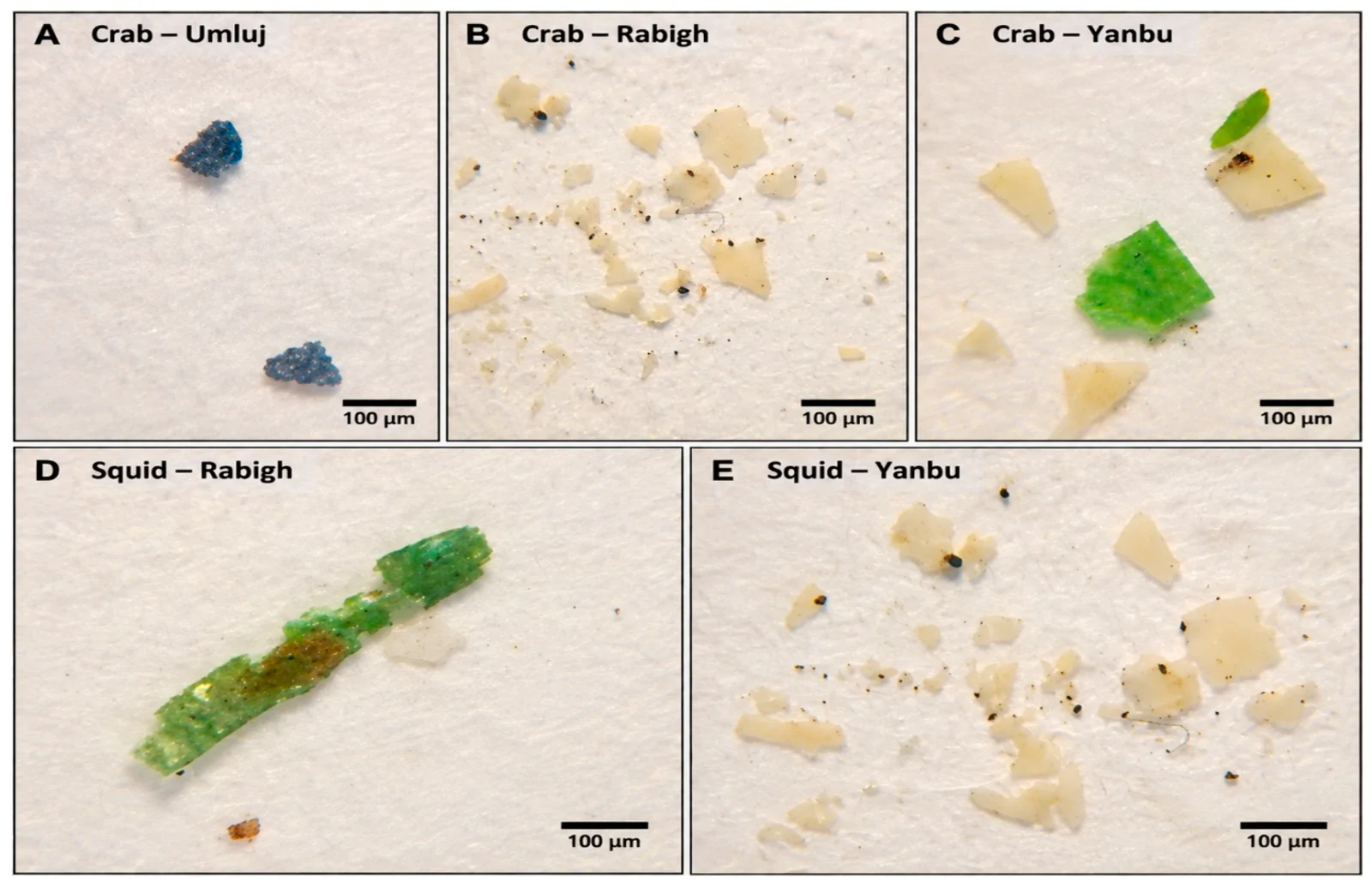
Representative microscopic images of particles recovered from crab and squid samples collected from Umluj, Rabigh, and Yanbu. Panels show (**A**) Crab-Umluj, (**B**) Crab-Rabigh, (**C**) Crab-Yanbu, (**D**) Squid-Rabigh, and (**E**) Squid-Yanbu. Scale bar = 100 µm.

FTIR examination was performed on all 508 microscopically identified suspected particles, of which 260 (51.2%) were spectroscopically confirmed as PE, while 248 (48.8%) remained unconfirmed. The PE spectra exhibited characteristic absorption bands, including CH_2_ stretching bands at approximately 2920 and 2850 cm^−1^ and CH_2_ rocking bands near 730 and 720 cm^−1^. A carbonyl band near 1740 cm^−1^ was also observed, consistent with weathering or oxidative modification of PE. Ten representative PE spectra are presented in Figure 2 to illustrate the characteristic spectral profiles. None of the particles that yielded interpretable FTIR spectra was morphologically classified as a fiber; therefore, the polymer identity of the visually classified fiber fraction was not established. Microscopic examination at 400× total magnification (WF10×/20 eyepiece and 40× objective; nominal field diameter approximately 500 µm) revealed fragments ranging from tens to low hundreds of micrometers. In the calibratable Crab-Rabigh field, the larger green fragment was estimated to be approximately 80–110 µm along its longest dimension, whereas the smaller fragment was estimated to be approximately 35–55 µm (Figure 3). These ranges reflect uncertainty associated with image-based calibration and are therefore presented as approximate size estimates rather than precise measurements. These size estimates describe representative visible particles rather than a population-level size distribution, because some images were cropped and did not include the full calibrated field of view. Accordingly, visually classified fibers are conservatively reported as suspected anthropogenic fibers rather than as confirmed microplastics.

### 3.3. Potentially Toxic Element Concentrations in Edible Muscle

PTE concentrations showed clear species-specific patterns (Table 4). Crab muscle contained comparatively higher levels of Zn, total As, and Cu, whereas squid muscle had substantially higher levels of Fe and Cd. In crabs, Zn increased from 1.29 ± 0.35 mg/kg ww at Umluj to 1.72 ± 0.73 mg/kg ww at Rabigh and 2.39 ± 0.45 mg/kg ww at Yanbu. Total As showed a similar descriptive spatial pattern, increasing from 0.63 ± 0.20 mg/kg ww at Umluj to 0.73 ± 0.35 mg/kg ww at Rabigh and 0.77 ± 0.21 mg/kg ww at Yanbu. In squid, Fe concentrations were comparatively high and stable among sites (4.29 ± 1.77 to 4.36 ± 1.02 mg/kg ww), while mean Cd concentrations were 0.42 ± 0.11 mg/kg ww at Umluj and 0.57 ± 0.24 and 0.57 ± 0.15 mg/kg ww at Rabigh and Yanbu, respectively. The measured Cd values remained below the applicable EU maximum levels of 0.50 mg/kg for crustaceans and 1.00 mg/kg for cephalopods; Pb and Hg were also below their applicable EU maximum levels in all species–site groups [46]. No directly applicable EU maximum levels were used for Zn, Fe, total As, Cu, Co, Ni, Mn, or Cr in this comparison. Across sites, squid contained approximately 17–24-fold more Fe and 5–7-fold more Cd than crabs, whereas crabs contained approximately 11–13-fold more total As and consistently higher Zn concentrations. Because specimen-level PTE data were unavailable for inferential analysis, these comparisons are descriptive, and no inferential *p*-values are reported.

**Figure 4:**
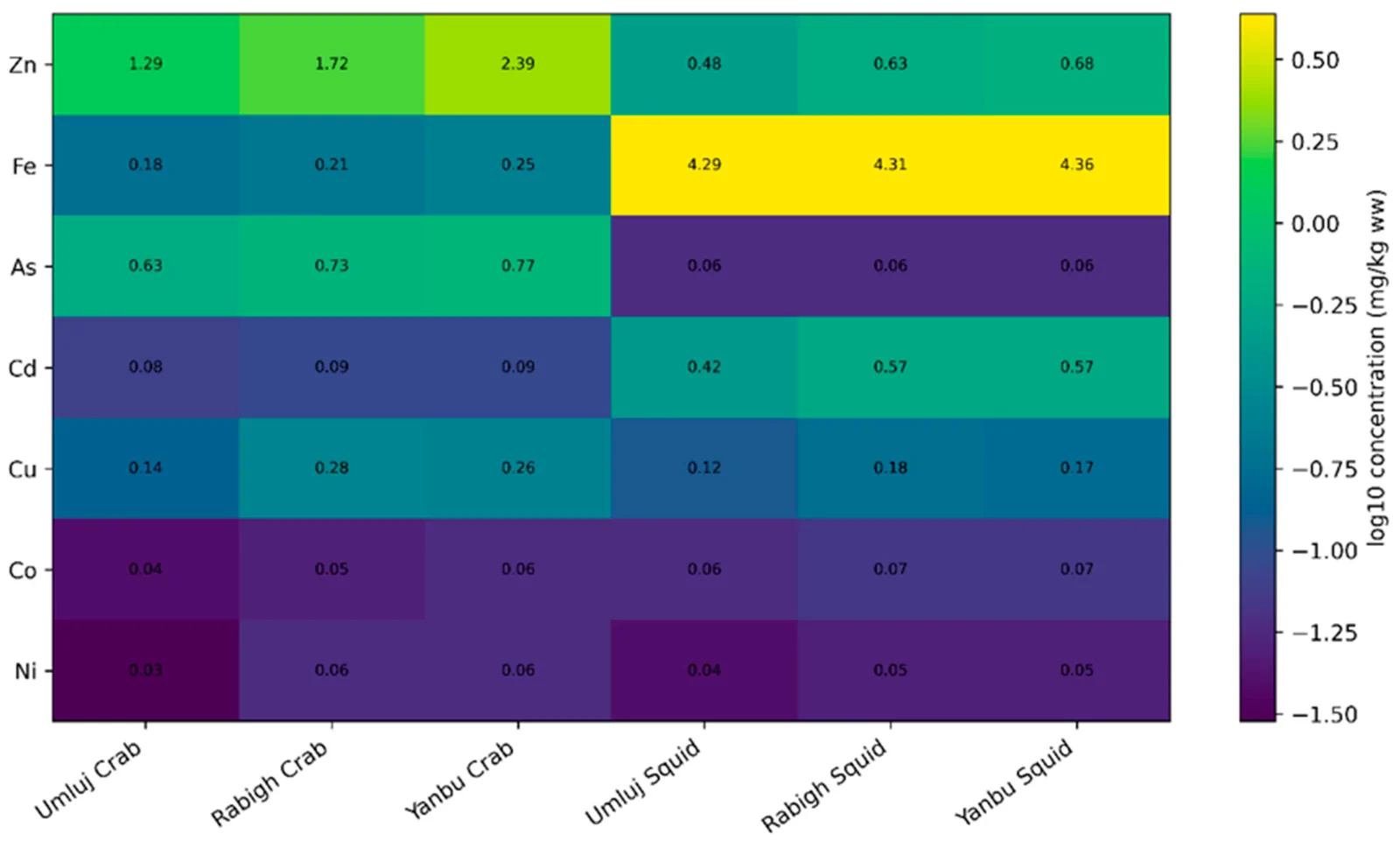
Heatmap of PTE concentrations in edible muscle of crab and squid from Umluj, Rabigh, and Yanbu, showing species- and site-specific accumulation patterns.

The heatmap further illustrates the contrasting elemental profiles of the two taxa (Figure 4). Crab samples were characterized primarily by relatively greater Zn and As concentrations, whereas squid samples were distinguished by substantially higher Fe and Cd concentrations. Site-related variation was less pronounced for several elements, particularly Fe in squid, which remained consistently high across the three sampling locations. These patterns correspond to the concentrations reported in Table 4.

### 3.4. Spatial and Species-Specific Variation in Metal Pollution Index

**Figure 5:**
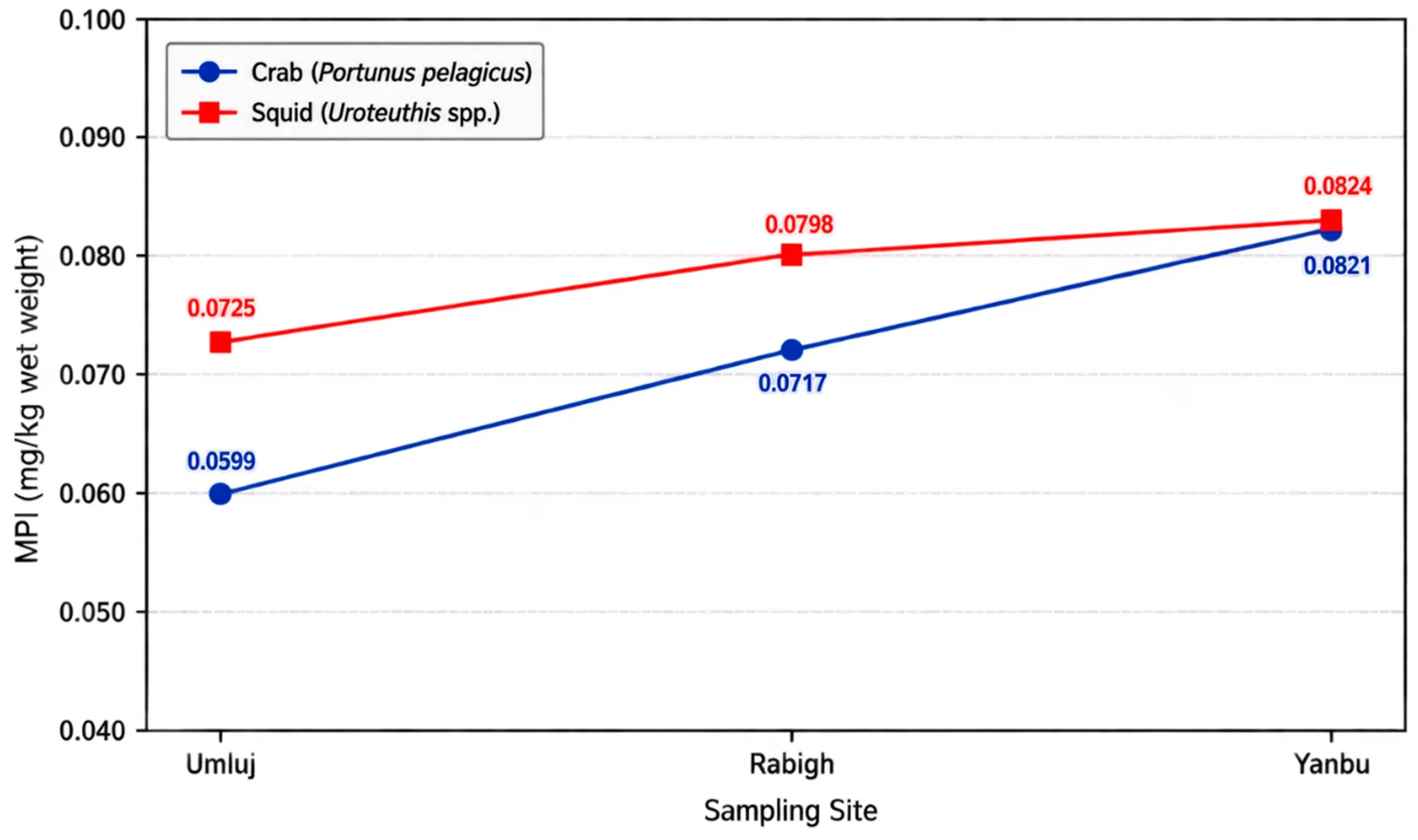
MPI for PTEs in edible muscle of Portunus pelagicus and Uroteuthis spp. collected from three Red Sea coastal sites. MPI was calculated as the geometric mean of the ten elements quantified numerically across all species–site combinations (Zn, Fe, As, Cd, Cu, Co, Ni, Mn, Pb, and Hg); Cr was excluded because its concentration in Umluj squid was below the limit of detection (<LOD). MPI is interpreted as a comparative measure of overall elemental burden rather than as a toxicological or regulatory risk threshold.

The MPI showed a consistent spatial increase in integrated elemental loading from Umluj to Yanbu in both species (Figure 5). In *Portunus pelagicus*, MPI increased from 0.0599 mg/kg ww at Umluj to 0.0717 mg/kg ww at Rabigh and 0.0821 mg/kg ww at Yanbu, corresponding to an approximately 37% increase between Umluj and Yanbu. In *Uroteuthis* spp., MPI increased more gradually from 0.0725 mg/kg ww at Umluj to 0.0798 mg/kg ww at Rabigh and 0.0824 mg/kg ww at Yanbu.

The highest MPI was recorded in Yanbu squid (0.0824 mg/kg ww), closely followed by Yanbu crab (0.0821 mg/kg ww), whereas Umluj crab had the lowest value (0.0599 mg/kg ww). Squid therefore exhibited a higher MPI than crab at Umluj and Rabigh, while values converged at Yanbu. This convergence occurred despite contrasting elemental profiles, with crabs characterized by relatively higher As and Zn and squid by substantially higher Fe and Cd. MPI values are interpreted as comparative indicators of integrated elemental burden rather than toxicological or regulatory risk thresholds.

### 3.5. Association Between Microplastic Abundance and PTE Concentrations

Because MPs and PTEs were quantified in different tissues (gill and digestive tissues versus edible muscle, respectively), the MP–PTE analysis can assess only organism-level covariation among the six species–site group means and cannot demonstrate transport of PTEs from MPs to muscle. Apparent group-level associations with individual elements were strongly influenced by the crab–squid contrast and are therefore treated as exploratory rather than confirmatory. Exploratory group-level analysis indicated strong separation by species. Apparent associations of MP abundance with individual PTE concentrations largely reflected the contrast between crab and squid rather than consistent within-species covariation. Species identity alone accounted for a large proportion of the group-level variation in MP abundance; however, with only six species–site group means, correlation and regression estimates are descriptive and should not be interpreted as robust inferential evidence. Within-species patterns were likewise uninformative because only three site means were available per species. Moreover, MPs and PTEs were measured in different tissues, precluding inference of MP-mediated PTE transfer.

### 3.6. Estimated Daily Intake of Potentially Toxic Elements

**Figure 6:**
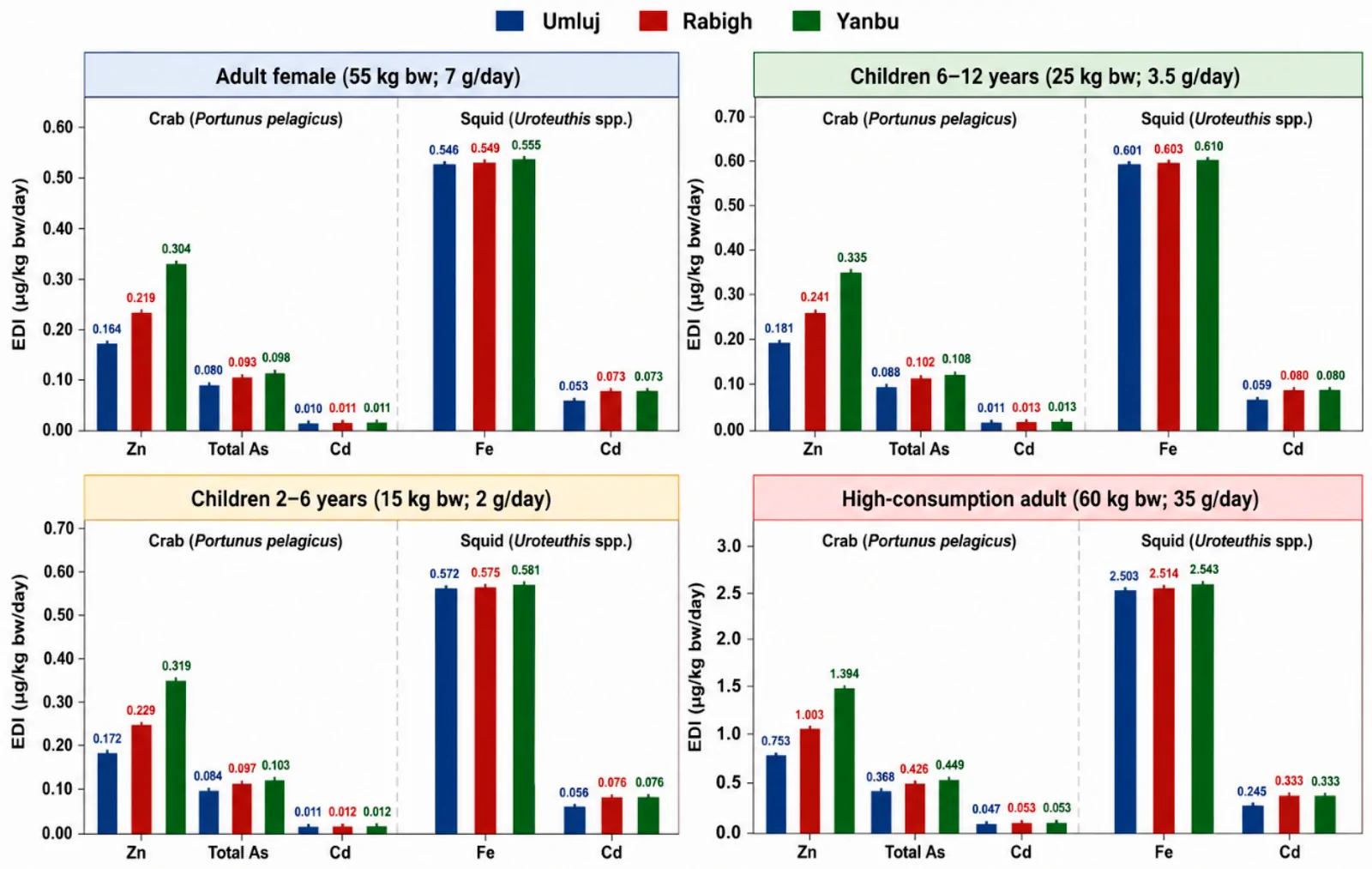
EDI (µg/kg bw/day) of selected PTEs from crab and squid across three Red Sea coastal sites under different exposure scenarios.

EDI varied according to species, element, sampling location, and consumer scenario (Table 5; Figure 6). Under the adult-female scenario, the principal exposures associated with crab consumption were Zn (0.164–0.304 µg/kg bw/day) and total As (0.080–0.098 µg/kg bw/day). Squid consumption resulted in substantially greater Fe (0.546–0.555 µg/kg bw/day) and Cd (0.053–0.073 µg/kg bw/day) exposure. Both child scenarios showed similar species- and element-specific patterns, with differences in absolute EDI values reflecting their respective food-ingestion-rate-to-body-weight ratios. The high-consumption adult scenario produced the highest EDI values. Squid Fe exposure ranged from 2.503 to 2.543 µg/kg bw/day, and Cd exposure ranged from 0.245 to 0.333 µg/kg bw/day. In contrast, crab consumption resulted in total-As EDI values of 0.368–0.449 µg/kg bw/day and Zn values of 0.753–1.394 µg/kg bw/day, with the highest crab exposures generally occurring at Yanbu. Overall, crab consumption was associated with higher Zn and total-As exposure, whereas squid consumption was associated with higher Fe and Cd exposure. EDI represents estimated dietary exposure rather than toxicological risk; the corresponding risk indices are therefore presented separately below.

### 3.7. Human Health Risk Assessment of PTEs

**Figure 7:**
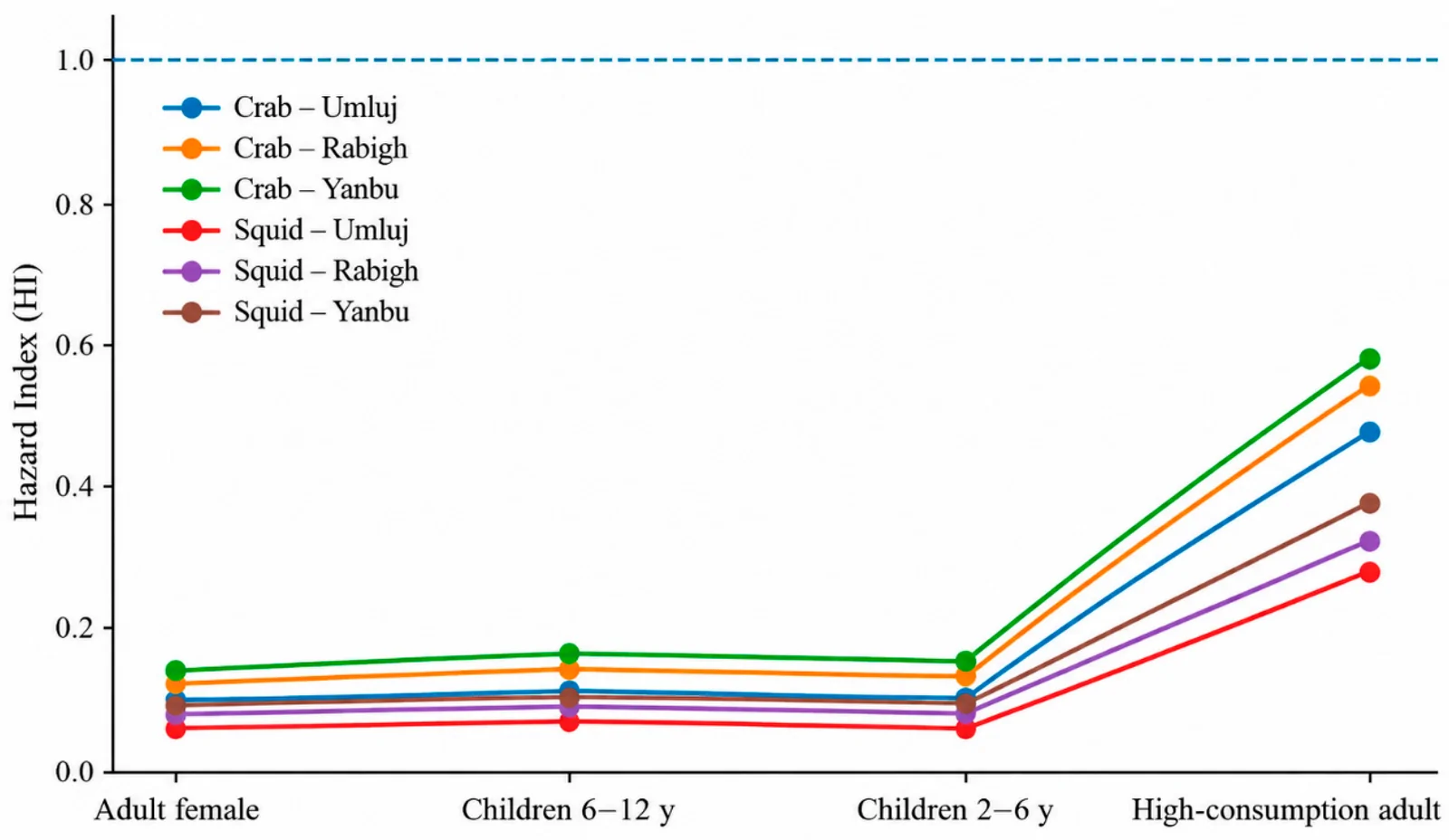
Hazard Index (HI) for cumulative non-carcinogenic exposure to iAs, Cd, and Cr through crab and squid consumption under four exposure scenarios.

HI values remained below 1.0 across species, sites, and exposure scenarios (Table S1; Figure 7). Under the adult-female scenario, HI ranged from 0.1037 to 0.1258 for crab and from 0.0624 to 0.0815 for squid. Among child consumers, the maximum HI values were 0.1384 for children aged 6–12 years and 0.1318 for children aged 2–6 years, both associated with Yanbu crab. The high-consumption adult scenario produced the highest non-carcinogenic risk estimates. Crab HI values were 0.4754, 0.5493, and 0.5765 at Umluj, Rabigh, and Yanbu, respectively, whereas corresponding squid values were 0.2858, 0.3733, and 0.3733. The maximum HI observed in the study was therefore 0.5765 for the Yanbu crab under the high-consumption scenario, which remained below the reference value of 1. Assumed inorganic arsenic (iAs) was the predominant contributor to crab HI, whereas Cd was the principal contributor to squid HI; Cr contributed negligibly.

**Figure 8:**
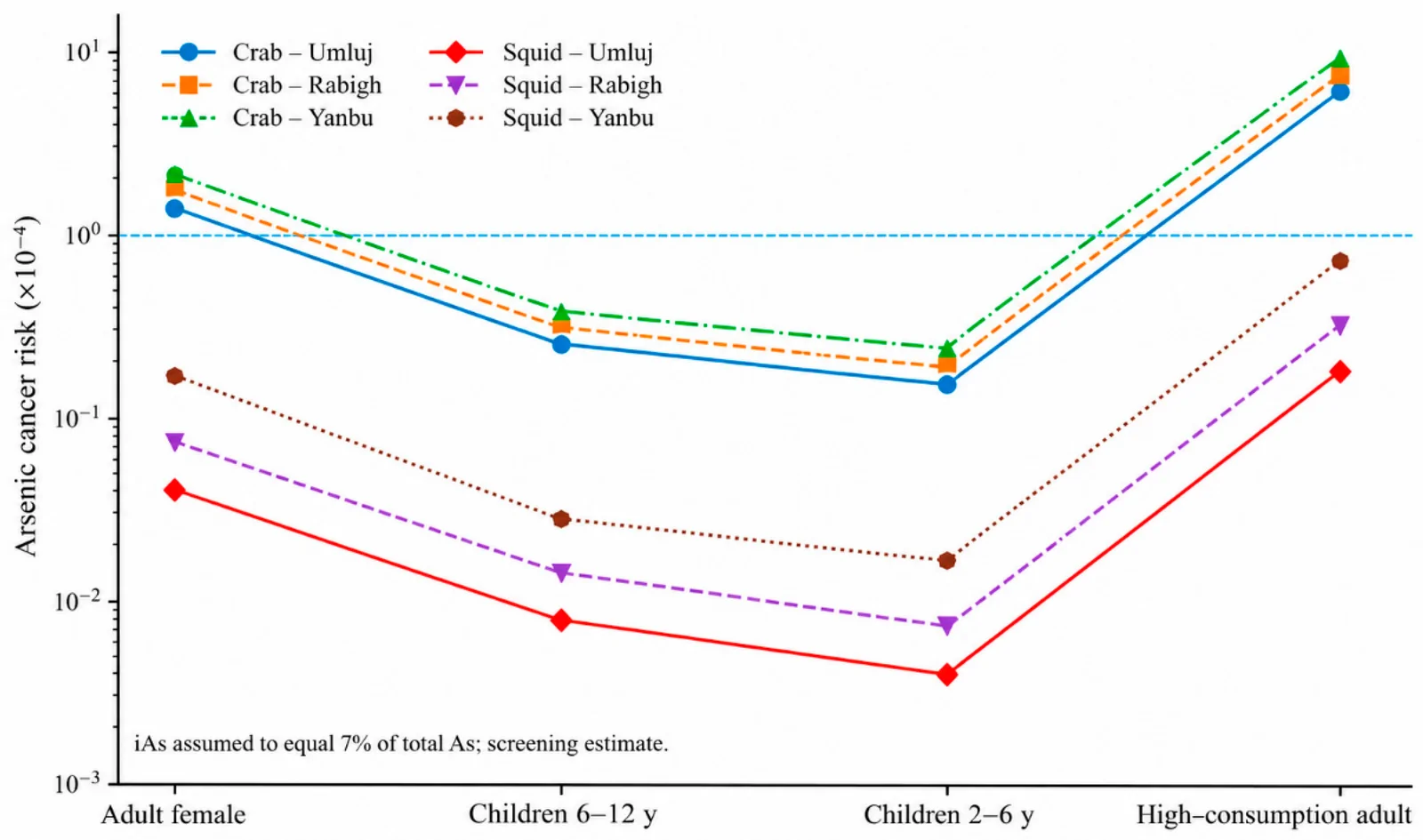
Estimated carcinogenic risk (CR) from dietary inorganic arsenic exposure through crab and squid consumption across the three sampling sites. iAs was assumed to represent 7% of total As.

CR estimates showed pronounced differences between species and exposure scenarios (Table S1; Figure 8). Based on the assumption that iAs represented 7% of measured total As, adult-female CR estimates for crab were 1.80 × 10^−4^ at Umluj, 2.08 × 10^−4^ at Rabigh, and 2.20 × 10^−4^ at Yanbu, compared with 1.71 × 10^−5^ for squid at all three sites. Lifetime-averaged estimates were lower for children because of their shorter exposure durations. For crab consumption, CR ranged from 2.82–3.45 × 10^−5^ in children aged 6–12 years and from 1.61–1.97 × 10^−5^ in children aged 2–6 years. The highest CR estimates occurred under the high-consumption adult scenario, reaching 8.23 × 10^−4^, 9.54 × 10^−4^, and 1.01 × 10^−3^ for Umluj, Rabigh, and Yanbu crab, respectively, compared with 7.84 × 10^−5^ for squid at all three sites. Thus, under the high-consumption scenario, Yanbu crab produced the highest modeled CR estimate. Because iAs was inferred as 7% of total As rather than measured directly by arsenic speciation, all CR values represent assumption-dependent screening estimates.

## 4. Discussion

This study provides an integrated assessment of MP contamination, PTE accumulation, and dietary health risks in two commercially important but ecologically contrasting seafood taxa from the Saudi Arabian Red Sea. Three findings are especially important. First, *Portunus pelagicus* showed substantially greater MP occurrence and burden than *Uroteuthis* spp., with crab burdens exceeding those of squid by approximately 10- to 89-fold depending on location. Second, MP abundance and PTE concentrations exhibited contrasting species-specific patterns: crab muscle contained substantially higher total As and Zn, whereas squid accumulated markedly higher Fe and Cd. Third, cumulative non-carcinogenic risk remained below the conventional threshold of concern (HI < 1) in all consumer scenarios, while carcinogenic-risk estimates for crabs were strongly dependent on the assumed iAs fraction and consumption rate [9]. These patterns extend previous seafood-contamination studies by demonstrating, within the same Red Sea sampling framework, that ecological traits, tissue-specific exposure, element-specific physiology, and risk-model assumptions can produce markedly different interpretations of contaminant burden and consumer risk [8,47,48,49,50,51].

### 4.1. Species- and Site-Specific Patterns of Microplastic Contamination

The most striking result was the magnitude of the interspecific difference in MP abundance. Mean total MP abundance in *Portunus pelagicus* ranged from 0.742 particles/g ww at Umluj to 1.683 particles/g ww at Yanbu, whereas *Uroteuthis* spp. contained only 0.008–0.167 particles/g ww. Crab burdens were therefore approximately 89-, 64-, and 10-fold higher than squid at Umluj, Rabigh, and Yanbu, respectively, with very large rank-biserial effect sizes (0.992–0.997). This separation is consistent with evidence that crab MP occurrence and body burden vary with habitat and functional traits and that feeding mode can influence MP burdens in wild crabs [3,42,52]. Bothma et al. quantified MPs in chokka squid (*Loligo reynaudii*) from the South Atlantic and Indian Oceans and found pronounced tissue and regional differences, including a possible growth-dilution effect [53]. Growth dilution therefore remains a plausible but untested hypothesis for the comparatively low MP abundance observed in *Uroteuthis* spp.; growth rate was not measured in the sampled squid, and the present data neither support nor refute this mechanism. Zhang et al. found MPs in crab guts and gills, with greater abundance and larger particle sizes in the guts, and no MPs were detected in muscle [42]. Together, these studies support the interpretation that benthic association, sediment contact, and feeding ecology can create exposure pathways that differ substantially from those of pelagic predatory squid. Finally, because particles retained in gill and digestive tissues are subject to ongoing ingestion, ventilation, and egestion, the counts reported here represent the occurrence of MPs at the time of capture rather than evidence of net long-term accumulation in these organs.

The present spatial pattern also agrees with the expectation that local anthropogenic pressure modifies exposure. Crab MP abundance was significantly higher at Rabigh and Yanbu than at Umluj, while squid showed its highest abundance at Yanbu. Yanbu and Rabigh are affected by industrial, shipping, urban, and desalination activities, whereas Umluj is comparatively less industrialized. However, this spatial pattern should not be interpreted as direct source attribution because hydrodynamic transport, wastewater, fishing activity, shoreline use, and sediment redistribution can all influence MP availability. Fibers were frequently observed among the morphologically classified particles; however, their polymer identity could not be confirmed by FTIR (Section 3.2). They are therefore conservatively reported as suspected anthropogenic fibers because visual identification alone cannot reliably establish polymer identity and may include both synthetic and non-synthetic materials [10,11,18]. Spectroscopic verification is therefore important for distinguishing visually suspected microplastics from other particles and for reliable polymer identification [10,18]. In the present study, all 508 microscopically identified suspected particles were examined by FTIR. Of these, 260 particles (51.2%) were spectroscopically confirmed as PE, whereas 248 particles (48.8%) remained unconfirmed. The identification of PE is consistent with its widespread occurrence in aquatic and marine biota [3,4,17]. However, the confirmed PE fraction should not be interpreted as representing the polymer composition of the entire particle population, because nearly half of the microscopically suspected particles could not be spectroscopically confirmed. Moreover, the visually classified fiber fraction remained unconfirmed. Accordingly, the FTIR findings provide evidence that PE was a substantial component of the recovered particles but do not establish that it was the only polymer present. This conservative interpretation is consistent with recommended microplastic characterization approaches that emphasize spectroscopic confirmation rather than reliance on visual morphology alone [10,11,18]. The frequent occurrence of fibers and the identification of PE are consistent with observations reported for marine invertebrates and other aquatic organisms [20,43,52,54]. Potential local sources include packaging materials, fishing gear, wastewater, and other coastal plastic inputs; however, source apportionment was beyond the scope of the present study design. The significantly greater MP abundance in crab digestive tissues than in gills across all three sites supports ingestion as an important exposure route, although tissue distribution is not universal. Experimental work in a predatory crab reported a tissue distribution of hepatopancreas > gut > gills > muscle after waterborne and trophic exposure [55], while field sampling of wild crabs found significantly greater abundance and larger particles in the guts than in the gills [42]. By contrast, the macro-ecotoxicological synthesis by Truchet et al. found substantial variability among tissues and species [52]. These differences reinforce the need to interpret organ-specific MP burdens in relation to feeding activity, particle characteristics, gut evacuation, ventilation, habitat, and analytical design, rather than treating any single tissue as a universal proxy for environmental MP contamination.

The relative contamination indices require similar caution. For crabs, CF increased from 1.00 at Umluj to 2.17 at Rabigh and 2.27 at Yanbu, yielding CD = 5.44 and an overall rPLI of 1.70. In squid, CF values were 1.00, 3.00, and 20.00 at Umluj, Rabigh, and Yanbu, respectively, yielding CD = 24.00 and rPLI = 3.91. The Yanbu CF of 20.00 was mathematically amplified by the extremely low Umluj reference concentration (0.0083 particles/g ww), even though the absolute difference was only 0.158 particles/g ww. Accordingly, CF, CD, and rPLI are useful here only as within-species relative descriptors; they should not be treated as validated ecological-risk thresholds or used to infer that squid were more polluted than crabs. This limitation is especially important when chemical-pollution index frameworks are transferred to sparse MP datasets containing values close to zero [19,52,55].

### 4.2. PTE Accumulation and Species-Specific Elemental Profiles

The elemental results, together with the exploratory group-level association analysis, indicate that the apparent correspondence between MP burden and PTE concentrations was strongly influenced by species identity; the limited group-level dataset does not support robust inference about within-species covariation. Crabs had far greater MP abundance, yet squid accumulated substantially more Fe and Cd, while crabs contained more total As and Zn. Across the six species–site groups, MP abundance primarily reflected the species contrast (crab > squid); because only six group means were available, the exploratory regression estimates are treated as descriptive rather than confirmatory. This contrast is consistent with the strong influence of taxonomy, physiology, feeding ecology, tissue distribution, and element-specific kinetics on seafood contaminant profiles [4,9,24,50,56,57]. The spatial increase in crab Zn and total As toward Yanbu and the higher integrated MPI at Yanbu indicate greater elemental burden in the sampled organisms at that location; however, the present design cannot attribute these patterns to a specific environmental, industrial, or urban source because water, sediment, prey, and discharge sources were not measured concurrently [9,58]. Importantly, MPI is an integrated comparative index, not a toxicological threshold.

### 4.3. Dietary Exposure and Human Health Implications

Microplastics can interact with PTEs through surface sorption, raising the possibility that ingested particles may act as vectors for co-contaminants in aquatic organisms [59,60,61,62,63]. However, the ecological importance of this pathway remains context-dependent. Experimental and review studies indicate that metal sorption and subsequent bioavailability are influenced by polymer properties, particle weathering, metal chemistry, and surrounding environmental conditions [60,61,62,63]. Importantly, naturally suspended particles and sediments may compete effectively with MPs for trace metals and, in some cases, exhibit greater adsorption capacity than synthetic polymers [60]. Moreover, gastrointestinal conditions can modify the desorption of particle-bound metals; acidic conditions may enhance release, whereas other gut conditions may favor continued association with the particle during passage [62,63,64]. Thus, the presence of both MPs and PTEs within an organism does not by itself demonstrate MP-mediated metal transfer.

This distinction is particularly relevant to the present study because MPs and PTEs were quantified in different tissues. MPs were assessed in gill and digestive tissues, whereas PTEs were quantified in edible muscle. Previous studies indicate that MPs are commonly retained in tissues directly involved in filtration and ingestion, particularly the gills and digestive compartments, whereas absorbed metals may cross physiological barriers and accumulate in internal organs via distinct uptake and distribution pathways [65,66,67,68]. Evidence from wild fish further indicates that MP translocation from gastrointestinal tissues to edible muscle can occur, but appears variable and does not necessarily follow simple trophic or bioaccumulation patterns [66]. Consequently, the exploratory MP–PTE relationships observed across the six species–site groups in the present study should be interpreted as organism-level covariation rather than evidence of direct transfer of PTEs from MPs to muscle. The lack of robust within-species covariation (Section 3.5) neither confirms nor excludes an MP-vector effect; resolving this mechanism would require paired specimen-level measurements, particle-associated metal analysis, and tissue-matched chemical measurements.

Life-history characteristics provide an additional explanation for variation in contaminant burdens. Age, somatic growth, metabolic turnover, feeding behavior, and contaminant-clearance rates can substantially modify contaminant concentrations in wild organisms [66,69,70]. Rapid biomass accumulation may produce a growth-dilution effect in which contaminant concentrations decrease on a mass-normalized basis, whereas longer-lived organisms may experience greater time-integrated exposure [66,69]. These processes could contribute to the pronounced differences observed between *Portunus pelagicus* and *Uroteuthis* spp.; however, individual age and growth rates were not measured in the present study. Growth dilution should therefore be regarded as a plausible biological hypothesis rather than a demonstrated explanation for the comparatively low MP burden in squid. Differences in species ecology, exposure routes, retention, elimination, and tissue distribution are also likely to contribute to the contrasting MP and PTE profiles. From a food-safety perspective, the quantitative risk assessment should be distinguished from these mechanistic considerations. The present assessment evaluates dietary exposure to PTEs through edible muscle using EDI, element-specific THQs, cumulative HI, and iAs-related CR; it does not quantify human health risk from MPs. All HI estimates remained below 1, including the high-consumption scenario, indicating that appreciable combined non-carcinogenic effects from the assessed PTEs are unlikely under the modeled exposure assumptions. The dominant contributors differed between taxa, with assumed iAs contributing most strongly to crab HI and Cd to squid HI. These findings reinforce the importance of species-specific contaminant profiles and consumption scenarios when evaluating seafood exposure. Carcinogenic-risk estimates require greater caution. Because arsenic speciation was not directly measured, iAs was assumed to represent 7% of total As. Consequently, CR values exceeding 1 × 10^−4^ in some adult crab scenarios are assumption-dependent screening estimates rather than direct evidence of carcinogenic exposure. Their magnitude depends on the assumed iAs fraction, consumption rate, body weight, exposure duration, and the applied cancer slope factor. Direct arsenic speciation is therefore necessary before definitive conclusions can be drawn regarding carcinogenic risk from crab consumption.

Finally, the MP findings should not be interpreted using the quantitative PTE risk framework. Although experimental evidence indicates that high MP exposure can affect intestinal integrity and gut microbial communities, such biological responses do not necessarily translate into increased systemic contaminant burdens [59,65,71]. The present MP data therefore establish occurrence and potential exposure, but not a quantitative human health risk. Future studies combining polymer-specific characterization, particle-associated PTE measurements, matched tissue chemistry, arsenic speciation, and individual age/size information would provide a stronger basis for determining whether MPs materially modify contaminant bioavailability and dietary risk in Red Sea seafood.

## 5. Strengths and Limitations

The principal strength of this study is the integrated comparison of two ecologically distinct commercial taxa across three Red Sea locations using a standardized MP subset and ICP-MS-based elemental analysis. Several limitations should nevertheless be considered. First, although FTIR analysis was performed on all 508 microscopically identified suspected particles, only 260 (51.2%) were spectroscopically confirmed as PE, while 248 (48.8%) remained unconfirmed. Consequently, total particle abundance is based on microscopic identification and should be interpreted as suspected-particle abundance, whereas polymer-specific conclusions are restricted to spectroscopically confirmed particles. The FTIR results demonstrate the occurrence of PE among the recovered particles but should not be extrapolated to the unconfirmed fraction or interpreted as evidence that PE was the only polymer present. In addition, the visually classified fiber fraction remained spectroscopically unconfirmed and is therefore conservatively reported as suspected anthropogenic fibers. Second, density separation with saturated NaCl (≈1.2 g/mL) may not efficiently recover denser polymers (e.g., PVC), and no formal polymer-specific recovery tests were performed. Third, although procedural blanks (*n* = 3) contained no suspected particles, the small number of blanks limits the ability to characterize rare background contamination, especially fibers. Fourth, the approximate 35–110 µm size estimates are derived from representative calibrated images and do not constitute a full particle-size distribution; a formal lower optical detection-size limit was not experimentally validated. Fifth, arsenic speciation was not measured, and iAs was inferred as 7% of total As. Sixth, although PTE concentrations are based on *n* = 30 specimens per species–site group, the specimen-level PTE dataset was unavailable for inferential reanalysis, preventing robust evaluation of species-by-site PTE effects or specimen-level MP–PTE associations. Seventh, specimen age could not be determined from commercial catches, so age-related effects on contaminant patterns remain unquantified. The exploratory MP–PTE association analysis was restricted to organism-level group means (*n* = 6), and MPs and PTEs were measured in different tissues. Finally, sampling covered September 2024 to January 2025 and did not include concurrent measurements of water, sediment, prey, or sources; seasonal variability and source attribution therefore remain unresolved. These constraints should guide interpretation of the present baseline and the design of future monitoring.

## 6. Conclusions

This study demonstrates clear species-specific differences in MP contamination and PTE accumulation in commercially important crab and squid from the Saudi Arabian Red Sea. Crabs showed substantially higher microscopy-based suspected-MP burdens than squid, particularly at Rabigh and Yanbu. All 508 microscopically identified suspected particles were examined by FTIR; 260 (51.2%) were spectroscopically confirmed as PE, whereas 248 (48.8%) remained unconfirmed, and the visually classified fiber fraction remained spectroscopically unconfirmed. PTE accumulation also differed markedly between species, with squid showing higher Fe and Cd and crabs higher total As and Zn. Non-carcinogenic dietary risk remained below the conventional threshold (HI < 1) across all evaluated scenarios, whereas arsenic-related carcinogenic-risk estimates were sensitive to the assumed inorganic-As fraction and consumption scenario. Exploratory MP–PTE associations were strongly influenced by species identity and do not demonstrate MP-mediated PTE transfer. Overall, these findings provide a baseline for Red Sea seafood contamination and highlight the need for expanded seasonal monitoring, direct arsenic speciation, and improved spectroscopic characterization of recovered particles.

## Figures and Tables

**Table 1:** Exposure parameters used for the non-carcinogenic and carcinogenic dietary risk assessment across consumer scenarios.

Consumer Scenario	BW (kg)	FIR (g/Day)	ED (Years)	EF (Days/Year)	ATc (Days)
Adult female	55	7.0	70	365	25,550
Children, 6–12 years	25	3.5	10	365	25,550
Children, 2–6 years	15	2.0	6	365	25,550
High-consumption adult	60	35.0	70	365	25,550

**Table 2:** Suspected-particle abundance, total particle counts, crab/squid fold differences, and contamination factors (CF) in the gill and digestive tissues of crabs and squid from three Red Sea sites.

Species	Site	Gill (Particles/g ww)	Digestive Tissue (Particles/g ww)	Combined Abundance (Particles/g ww)	Total Particles Counted	Crab/Squid Fold	CF
Crab	Umluj	0.350 ± 0.438	1.133 ± 0.524	0.742 ± 0.413	89	89	1.00
Crab	Rabigh	1.050 ± 0.547	2.167 ± 0.854	1.608 ± 0.659	193	64.3	2.17
Crab	Yanbu	1.067 ± 0.487	2.300 ± 0.783	1.683 ± 0.557	202	10.1	2.27
Squid	Umluj	0.000 ± 0.000	0.017 ± 0.091	0.008 ± 0.046	1	—	1.00
Squid	Rabigh	0.000 ± 0.000	0.050 ± 0.153	0.025 ± 0.076	3	—	3.00
Squid	Yanbu	0.033 ± 0.127	0.300 ± 0.407	0.167 ± 0.240	20	—	20.0

**Table 3:** Relative pollution load index (rPLI), contamination factor (CF), and degree of contamination (CD) for microplastics in crabs and squid from the Red Sea.

Species	CF (Umluj)	CF (Rabigh)	CF (Yanbu)	CD	rPLI	Classification
Crab	1.00	2.17	2.27	5.44	1.70	Elevated relative to the species-specific reference; descriptive only
Squid	1.00	3.00	20.0	24.0	3.91	Not classified—indices are mathematically inflated by the near-zero reference concentration (0.0083 particles/g ww); reported for descriptive transparency only

Note: CF, CD, and rPLI are used here only as species-normalized descriptive indices of relative spatial loading. Because each species is referenced to its own lowest mean abundance, these values should not be interpreted as validated ecological-risk categories or used for direct comparison of absolute contamination between crab and squid. The very low Umluj squid reference concentration (0.0083 particles/g ww) mathematically inflates the corresponding squid CF and rPLI values.

**Table 4:** Mean ± SD concentrations of potentially toxic elements (mg/kg wet weight) in edible muscle of crabs and squid from three Red Sea sites, with applicable EU maximum levels for Cd, Pb, and Hg. Values are based on *n* = 30 specimens per species–site group.

Element	Umluj Crab	Rabigh Crab	Yanbu Crab	Umluj Squid	Rabigh Squid	Yanbu Squid	Applicable EU Maximum Level/Regulatory Note
Zn	1.29 ± 0.35	1.72 ± 0.73	2.39 ± 0.45	0.48 ± 0.26	0.63 ± 0.32	0.68 ± 0.20	No directly applicable EU maximum level used
Fe	0.18 ± 0.11	0.21 ± 0.12	0.25 ± 0.08	4.29 ± 1.77	4.31 ± 1.55	4.36 ± 1.02	No directly applicable EU maximum level used
As	0.63 ± 0.20	0.73 ± 0.35	0.77 ± 0.21	0.0 6 ± 0.03	0.06 ± 0.03	0.06 ± 0.02	No directly applicable EU maximum level used
Cd	0.08 ± 0.12	0.09 ± 0.23	0.09 ± 0.03	0.42 ± 0.11	0.57 ± 0.24	0.57 ± 0.15	0.50 mg/kg (crab); 1.00 mg/kg (squid)
Cu	0.14 ± 0.05	0.28 ± 0.61	0.26 ± 0.09	0.12 ± 0.07	0.18 ± 0.09	0.17 ± 0.07	No directly applicable EU maximum level used
Co	0.04 ± 0.02	0.05 ± 0.03	0.06 ± 0.02	0.06 ± 0.02	0.07 ± 0.02	0.07 ± 0.02	No directly applicable EU maximum level used
Ni	0.03 ± 0.02	0.06 ± 0.03	0.06 ± 0.02	0.04 ± 0.02	0.05 ± 0.03	0.05 ± 0.02	No directly applicable EU maximum level used
Mn	0.05 ± 0.03	0.06 ± 0.03	0.06 ± 0.02	0.03 ± 0.01	0.06 ± 0.03	0.06 ± 0.02	No directly applicable EU maximum level used
Cr	0.03 ± 0.01	0.04 ± 0.01	0.04 ± 0.01	<LOD	0.02 ± 0.01	0.003 ± 0.0002	No directly applicable EU maximum level used
Pb	0.003 ± 0.002	0.003 ± 0.002	0.003 ± 0.004	0.003 ± 0.002	0.003 ± 0.002	0.002 ± 0.0004	0.50 mg/kg (crab); 0.30 mg/kg (squid)
Hg	0.002 ± 0.0003	0.001 ± 0.0004	0.002 ± 0.0004	0.003 ± 0.0003	0.001 ± 0.0004	0.002 ± 0.0005	0.50 mg/kg (crab); 0.30 mg/kg (squid)

**Table 5:** Key EDI values (µg/kg bw/day) for PTEs from consumption of crab and squid under different exposure scenarios.

Consumer Scenario	Species	Zn	Fe	Total As	Cd
Adult female	Crab	0.164–0.304	0.023–0.032	0.080–0.098	0.010–0.011
	Squid	0.061–0.087	0.546–0.555	0.008	0.053–0.073
Children 6–12 y	Crab	0.181–0.335	0.025–0.035	0.088–0.108	0.011–0.013
	Squid	0.067–0.095	0.601–0.610	0.008	0.059–0.080
Children 2–6 y	Crab	0.172–0.319	0.024–0.033	0.084–0.103	0.011–0.012
	Squid	0.064–0.091	0.572–0.581	0.008	0.056–0.076
High-consumption adult	Crab	0.753–1.394	0.105–0.146	0.368–0.449	0.047–0.053
	Squid	0.280–0.397	2.503–2.543	0.035	0.245–0.333

## Data Availability

The data supporting the findings of this study are available from the corresponding author upon reasonable request.

## Supplementary Materials

The following supporting information can be downloaded at: https://www.mdpi.com/article/10.3390/toxics14100888/s1, Table S1 contains recalculated non-carcinogenic and carcinogenic screening indices for four consumer exposure scenarios.
