# Supplementary material for: Occurrence of Microplastics and Potentially Toxic Elements in Red Sea Crab (Portunus pelagicus) and Squid (Uroteuthis spp.): Contamination and Food-Safety Concerns
Source: Toxics. 2026 Oct 2;14(10):888. doi: 10.3390/toxics14100888 (PMC13709861; doi:10.3390/toxics14100888)
Supplement: Supplementary file 1 [file toxics-14-00888-s001.zip › toxics-4584132-supplementary.pdf]

Supplementary Material

Occurrence of Microplastics and Potentially Toxic Elements in Red Sea Crab *Portunus pelagicus* and Squid *Uroteuthis* spp.: Contamination and Food Safety Concerns

Omar M. L. Alharbi

Table S1. Recalculated non-carcinogenic and carcinogenic screening indices for four consumer exposure scenarios (Supplementary Materials). Note: iAs was assumed to equal 7% of total As. HI is the sum of THQs for iAs, Cd, and Cr. CR was calculated using the fixed 70-year lifetime averaging time (25,550 days) and is therefore an assumption-dependent screening estimate.

Table S1. *Non-carcinogenic and carcinogenic dietary-risk screening indices for crab and squid from Umluj, Rabigh, and Yanbu under four consumer exposure scenarios.*

Species	Site	Consumer scenario	THQ iAs	THQ Cd	THQ Cr	HI	CR	Interpretation
**Crab**	Umluj	Adult female	0.0935	0.0102	0.000003	0.1037	1.80×10^{-4}	HI < 1
**Crab**	Umluj	Children 6–12 years	0.1029	0.0112	0.000003	0.1141	2.82×10^{-5}	HI < 1
**Crab**	Umluj	Children 2–6 years	0.0980	0.0107	0.000003	0.1087	1.61×10^{-5}	HI < 1
**Crab**	Umluj	High-consumption adult	0.4288	0.0467	0.000012	0.4754	8.23×10^{-4}	HI < 1
**Crab**	Rabigh	Adult female	0.1084	0.0115	0.000003	0.1199	2.08×10^{-4}	HI < 1
**Crab**	Rabigh	Children 6–12 years	0.1192	0.0126	0.000004	0.1318	3.27×10^{-5}	HI < 1
**Crab**	Rabigh	Children 2–6 years	0.1136	0.0120	0.000004	0.1256	1.87×10^{-5}	HI < 1
**Crab**	Rabigh	High-consumption adult	0.4968	0.0525	0.000016	0.5493	9.54×10^{-4}	HI < 1
**Crab**	Yanbu	Adult female	0.1143	0.0115	0.000003	0.1258	2.20×10^{-4}	HI < 1
**Crab**	Yanbu	Children 6–12 years	0.1258	0.0126	0.000004	0.1384	3.45×10^{-5}	HI < 1

**Crab**	Yanbu	Children 2–6 years	0.1198	0.0120	0.000004	0.1318	1.97×10^{-5}	HI < 1
**Crab**	Yanbu	High-consumption adult	0.5240	0.0525	0.000016	0.5765	1.01×10^{-3}	HI < 1
**Squid**	Umluj	Adult female	0.0089	0.0535	0.000000	0.0624	1.71×10^{-5}	HI < 1
**Squid**	Umluj	Children 6–12 years	0.0098	0.0588	0.000000	0.0686	2.69×10^{-6}	HI < 1
**Squid**	Umluj	Children 2–6 years	0.0093	0.0560	0.000000	0.0653	1.54×10^{-6}	HI < 1
**Squid**	Umluj	High-consumption adult	0.0408	0.2450	0.000000	0.2858	7.84×10^{-5}	HI < 1
**Squid**	Rabigh	Adult female	0.0089	0.0725	0.000002	0.0815	1.71×10^{-5}	HI < 1
**Squid**	Rabigh	Children 6–12 years	0.0098	0.0798	0.000002	0.0896	2.69×10^{-6}	HI < 1
**Squid**	Rabigh	Children 2–6 years	0.0093	0.0760	0.000002	0.0853	1.54×10^{-6}	HI < 1
**Squid**	Rabigh	High-consumption adult	0.0408	0.3325	0.000008	0.3733	7.84×10^{-5}	HI < 1
**Squid**	Yanbu	Adult female	0.0089	0.0725	0.000000	0.0815	1.71×10^{-5}	HI < 1
**Squid**	Yanbu	Children 6–12 years	0.0098	0.0798	0.000000	0.0896	2.69×10^{-6}	HI < 1
**Squid**	Yanbu	Children 2–6 years	0.0093	0.0760	0.000000	0.0853	1.54×10^{-6}	HI < 1
**Squid**	Yanbu	High-consumption adult	0.0408	0.3325	0.000000	0.3733	7.84×10^{-5}	HI < 1

Notes. *THQ = target hazard quotient; HI = hazard index; CR = carcinogenic risk; iAs = inorganic arsenic. Inorganic arsenic was estimated to be 7% of the measured total arsenic because arsenic speciation was not directly measured. For chromium measurements reported as <LOD, a concentration of zero was used in the deterministic screening calculation. CR values are assumption-dependent screening estimates. HI < 1 indicates a low likelihood of appreciable combined non-carcinogenic effects under the modeled assumptions. Exposure scenarios: adult female (BW = 55 kg; FIR = 7.0 g/day; ED = 70 years); children aged 6–12 years (BW = 25 kg; FIR = 3.5 g/day; ED = 10 years); children aged 2–6 years (BW = 15 kg; FIR = 2.0 g/day; ED = 6 years); and high-*

consumption adult (BW = 60 kg; FIR = 35.0 g/day; ED = 70 years). Exposure frequency was 365 days/year. For carcinogenic risk, a fixed lifetime averaging time of 25,550 days was applied to all groups.
